# Spontaneous Coronary Artery Dissection (SCAD) in an Atypical Patient Without Risk Factors and Prior Asymptomatic COVID-19 Infection

**DOI:** 10.7759/cureus.40906

**Published:** 2023-06-24

**Authors:** Neal Shah, Neehar Shah, Samir Mehta, Ellen Murray, Anna Grodzinsky

**Affiliations:** 1 Internal Medicine, University of Missouri Kansas City School of Medicine, Kansas City, USA; 2 Cardiology, Saint Luke's Mid America Heart Institute, Kansas City, USA

**Keywords:** cath lab, st-elevation myocardial infarction (stemi), covid-19 and heart, spontaneous coronary dissection, covid 19

## Abstract

Spontaneous coronary artery dissection (SCAD) is a condition primarily seen in young women and is characterized by non-atherosclerotic arterial damage. It can occur with or without conventional risk factors for coronary heart disease and is often associated with chronic inflammatory conditions. Here, we present a unique instance of a 67-year-old woman without known risk factors who developed sudden onset chest pain in the setting of an asymptomatic coronavirus 2019 (COVID-19) infection three weeks earlier. Subsequent evaluation revealed SCAD in the distal left anterior descending (LAD) artery.

## Introduction

Spontaneous coronary artery dissection (SCAD) is a non-atherosclerotic condition predominantly observed in young women with or without conventional risks for coronary heart disease [1]. Common risk factors are associated with chronic inflammatory conditions and vessel wall damage. Some examples include fibromuscular dysplasia, pregnancy/postpartum status, connective tissue disorders, severe hypertension, and drug use, among others. While the cardiovascular manifestations of the coronavirus 2019 (COVID-19) virus have been comprehensively described, there is limited published literature linking SCAD and COVID-19. In this report, we describe a unique case of SCAD in a patient without prior risk factors, occurring after the resolution of an asymptomatic COVID-19 infection. 

This article was previously presented at the 2022 American College of Physicians Internal Medicine Meeting on April 30th, 2022 and at the 2022 Society of General Internal Medicine Meeting on April 8th, 2022. 

## Case presentation

A 67-year-old woman with no known past medical history presented after acute onset chest pain while watching TV. The pain was described as a non-radiating, burning, substernal pain associated with shortness of breath and nausea. She had no prior history of similar chest pain and was recently exercising with no complaints. Her pain was not relieved by Tums, so she presented to the ED. She was hemodynamically stable on presentation and physical examination was unremarkable. A COVID-PCR test was positive on admission, however, the patient stated she had an incidental exposure at work three weeks prior and was asymptomatic during the time preceding her presentation. She routinely followed up with her primary care physician and never had chest pain prior to the presentation. Prior fasting lipid panels were normal. Social history was negative for tobacco use, alcohol, or other drugs. Her family history was remarkable for the myocardial infarction in her mother.

**Figure 1:**
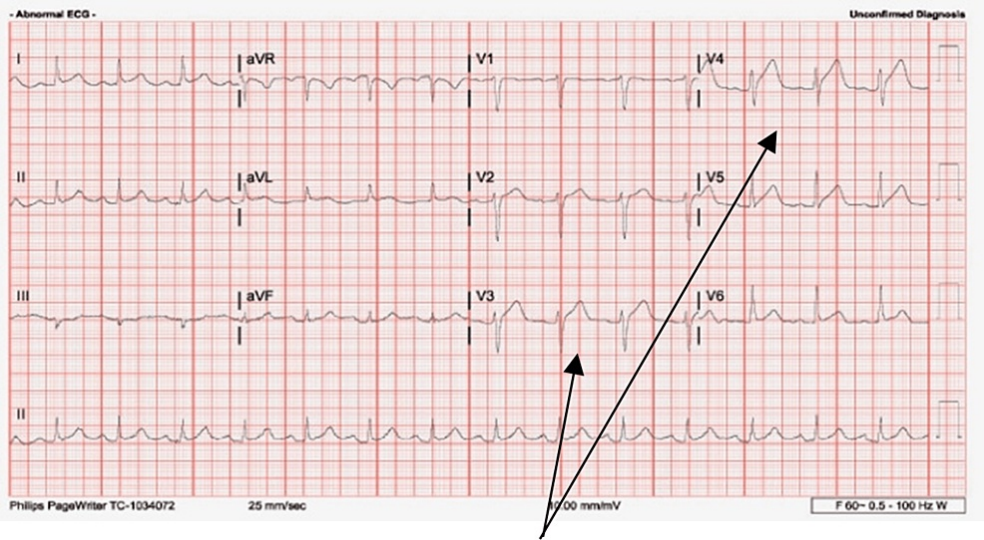
Initial ECG obtained at presentation demonstrating anterolateral STEMI. The black arrows mark the ST-elevations. ECG, electrocardiogram

**Figure 2:**
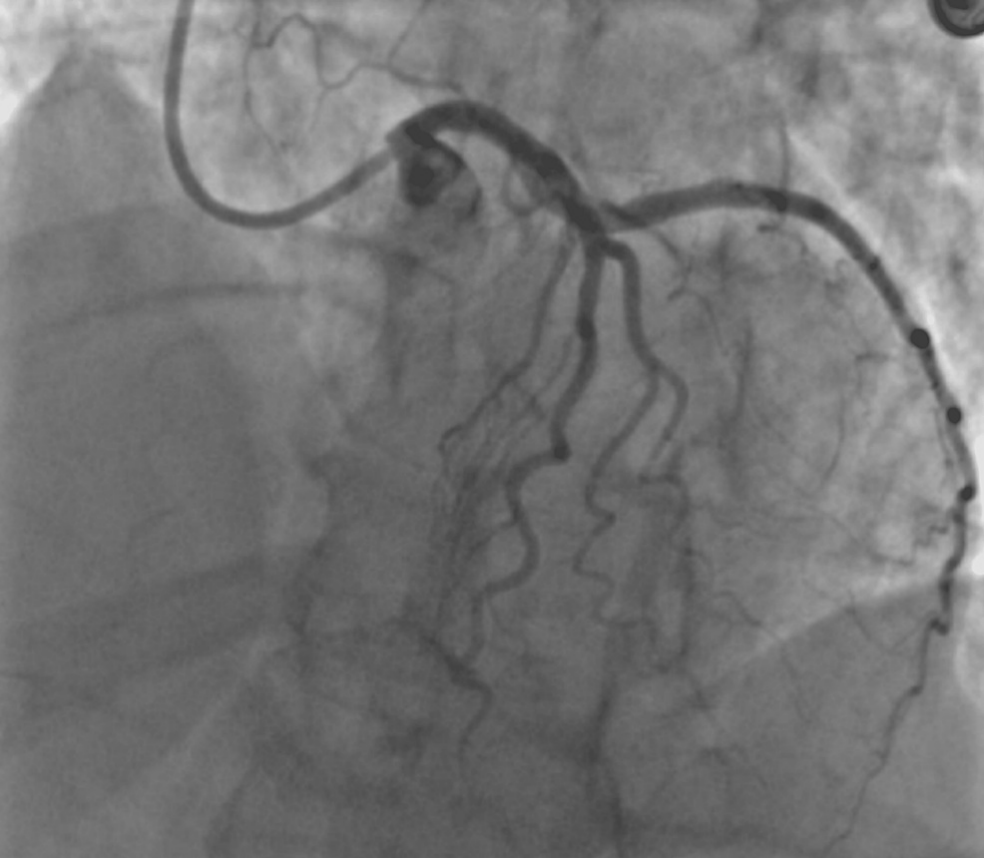
Distal apical SCAD of LAD viewed upon angiography. SCAD, spontaneous coronary artery dissection; LAD, left anterior descending

She was given sublingual nitroglycerin 0.4 mg which improved her pain. Electrocardiogram (ECG) demonstrated ST elevations in leads V3 and V4 with an initial troponin of 0.1 ng/mL (reference range <0.80 ng/mL). Repeat ECG revealed persistent ST-segment elevation in leads V3 and V4 (Figure 1). The N-terminal pro-B-type natriuretic peptide was 18 pg/mL (normal 0-100 pg/mL). Troponin peaked at 8.10 ng/mL (reference range <0.80 ng/mL) and trended down on further testing. C-reactive protein was 24 mg/L (reference range <10 mg/L). Complete blood count, basic metabolic panel, fasting lipid panel, and hemoglobin A1C were all normal. She subsequently received ticagrelor 180 mg, aspirin 324 mg, and heparin bolus of 4000u, and was taken for urgent cardiac catheterization. Left heart catheterization demonstrated distal left anterior descending (LAD) SCAD (Figure 2).

**Figure 3:**
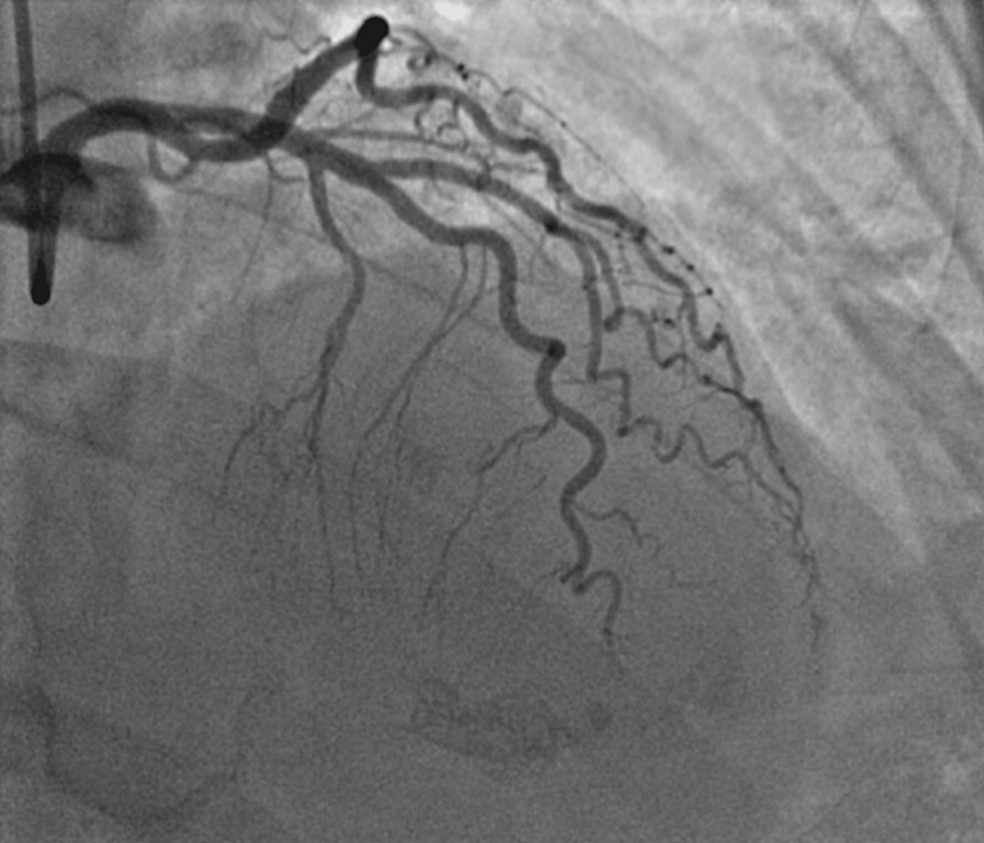
Post POBA: repeat angiographic imaging of LAD demonstrating restoration of flow. POBA, plain old balloon angioplasty; LAD, left anterior descending

She underwent plain old balloon angioplasty (POBA) with good restoration of vessel flow (Figure 3). Echocardiogram was obtained post-procedure and demonstrated an ejection fraction of 65% with apical akinesis. She was started on metoprolol succinate 25 mg daily, atorvastatin 80 mg daily, and continued aspirin 81 mg daily. Initiation of ace-inhibitor was deferred to outpatient due to normal blood pressure throughout admission. The patient was subsequently discharged in stable condition.

Our patient was seen for a one-month outpatient follow-up. Since the hospitalization, she remained asymptomatic with no reoccurrence of chest pain. She was also transitioned to Carvedilol 3.125 mg twice daily (Table 1).

**Table 1:** Timeline of presentation. COVID-19, coronavirus 2019; ECG, electrocardiogram; STEMI, ST-elevation myocardial infarction; LAD, left anterior descending; SCAD, spontaneous coronary artery dissection; POBA, plain old balloon angioplasty

Timeline	Patient history
Three weeks prior	A 67-year-old woman is exposed to COVID-19 at work. She remains asymptomatic in the time preceding admission
Day 1	Presents with chest pain and shortness of breath. COVID-19 positive on admission
Day 1	ECG confirms STEMI
	Taken to the cath lab and distal LAD SCAD was diagnosed. She underwent POBA with the restoration of flow
Day 2	Resting comfortably post-op. Echocardiogram demonstrates an ejection fraction of 65% with apical akinesis. She was started on aspirin, statin, and beta-blocker
Day 3	Discharged
Day 27	Recovered well with no reoccurrence of chest pain at follow-up

## Discussion

**Figure 4:**
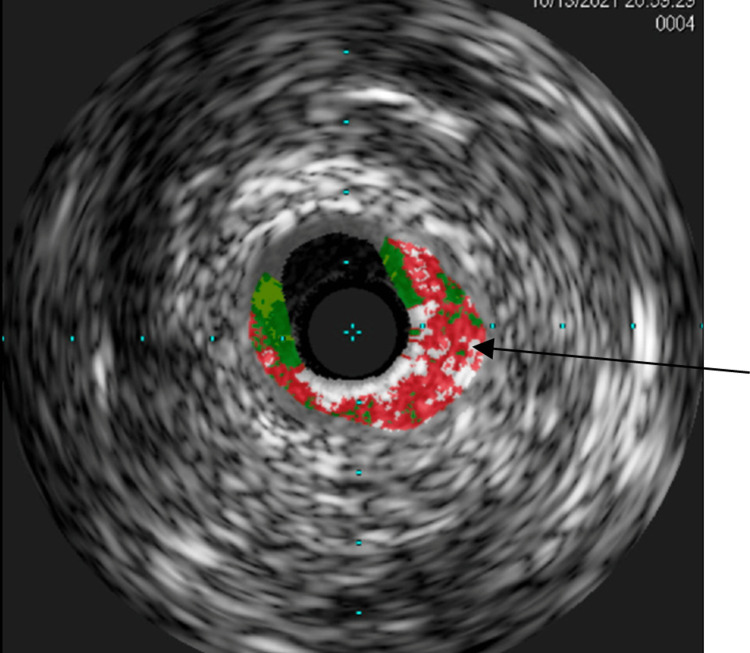
Dissection shown with IVUS imaging of the LAD. The red blood cells extravasating outside of the lumen of the vessel are represented with the black arrow. IVUS, intravascular ultrasound; LAD, left anterior descending

Patients with SCAD commonly present with typical chest pain and exhibit dynamic ECG changes and/or elevated biomarkers indicative of acute coronary syndrome. In some cases, patients may have concomitant conditions such as fibromuscular dysplasia, pregnancy/postpartum status, or connective tissue diseases. The diagnosis of SCAD is made by coronary angiogram, at times complemented by intravascular ultrasound or optical coherence tomography for further visualization (Figure 4). Sometimes, patients with SCAD may also have associated conditions such as fibromuscular dysplasia, pregnancy/postpartum status, or connective tissue diseases [1-2]. We describe a unique case of SCAD in a patient without any known risk factors, which occurred subsequent to the resolution of an asymptomatic COVID-19 infection. 

Extensive literature had discussed the cardiac complications associated with COVID-19, including myocarditis, myocardial infarction, heart failure, endotheliitis, and arrhythmias. However, the association between COVID-19 and SCAD has been sparsely reported, with only six studies documenting a potential link (Table 2) [3-5, 6-8]. In the majority of these cases, patients were acutely symptomatic with COVID-19 and subsequently developed angina during the hospitalization [4-6, 8]. Two similar cases reported SCAD occurring three months after the resolution of a prior COVID-19 infection [3, 7]. Notably, one of these patients had a history of symptomatic COVID-19 infection, while the case presented here involves an asymptomatic COVID infection [3]. Additionally, the other case involved a patient with preexisting cardiac risk factors that may have contributed to the development of SCAD [7]. 

**Table 2:** Summary of prior reports with this case represented in bold. CAD, coronary artery dissection

Case	Sex	Cardiac Hx (CAD risks)	Timing of chest pain and COVID-19 infection
Cannata et al. 2020 [3]	48 y/o F	No	Prior symptomatic covid - presented with chest pain weeks later
Albiero et al. 2020 [6]	70 y/o M	Yes	Symptomatic covid and chest pain on presentation
Fernandez et al. 2020 [8]	39 y/o M	No	Respiratory failure on presentation - developed subsequent chest pain
Papanikolaou et al. 2021 [5]	51 y/o F	Yes	Respiratory failure on presentation - developed subsequent chest pain
Courand et al. 2020 [4]	55 y/o M	Yes	Symptomatic covid on presentation - developed subsequent chest pain
Kumar et al. 2021 [7]	48 y/o F	Yes	Prior asymptomatic covid - presented with chest pain weeks later
Our case	67 y/o F	No	Prior asymptomatic covid - presented with chest pain weeks later
	3M, 4F	4/7 cardiac hx	4/7 symptomatic on presentation

Spontaneous coronary artery dissection is commonly associated with chronic inflammatory diseases that weaken the vessel wall [9]. In this particular case, the patient had no history of inflammatory conditions or cardiovascular risk factors that could contribute to SCAD development. However, it is established that COVID-19 triggers a marked inflammatory and immune response during infection, which can damage endothelial and smooth muscle cells in blood vessels [10]. It is plausible the inflammatory response induced by the infection could promote the fragility of coronary vessels and lead to SCAD in this patient. A limitation, in this case, is the inability to rule out an idiopathic origin of SCAD [11]. As such, further work should be conducted to elucidate the pathogenesis between COVID-19 and SCAD.

## Conclusions

As the relationship between SCAD and COVID-19 continues to be explored, it is crucial for providers to be mindful of the potential cardiac manifestations associated with the virus. The presence or absence of COVID-19 symptoms does not correlate with the emergence of cardiac complications. Furthermore, the development of SCAD can occur in individuals without prior cardiac risk factors or chronic inflammatory conditions. In consequence, it is essential to maintain a high index of suspicion for SCAD in patients presenting with COVID-19 or those with a history of COVID-19, regardless of their prior risk factors. 
